# Fabrication and Wettability Study of WO_3_ Coated Photocatalytic Membrane for Oil-Water Separation: A Comparative Study with ZnO Coated Membrane

**DOI:** 10.1038/s41598-017-01959-y

**Published:** 2017-05-10

**Authors:** Mohammed A. Gondal, Muhammad S. Sadullah, Talal F. Qahtan, Mohamed A. Dastageer, Umair Baig, Gareth H. McKinley

**Affiliations:** 1 Laser Research Group, Physics Department and Center of Excellence in Nanotechnology, King Fahd University of Petroleum & Minerals, Dhahran, 31261 Saudi Arabia; 2 Center of Excellence for Scientific Research Collaboration with MIT, King Fahd University of Petroleum and Minerals, Dhahran, 31261 Saudi Arabia; 3 Department of Mechanical Engineering, Massachusetts Institute of Technology, Cambridge, Massachusetts 02139-4307 United States

## Abstract

Superhydrophilic and underwater superoleophobic surfaces were fabricated by facile spray coating of nanostructured WO_3_ on stainless steel meshes and compared its performance in oil–water separation with ZnO coated meshes. The gravity driven oil-water separation system was designed using these surfaces as the separation media and it was noticed that WO_3_ coated stainless steel mesh showed high separation efficiency (99%), with pore size as high as 150 µm, whereas ZnO coated surfaces failed in the process of oil-water separation when the pore exceeded 50 µm size. Since, nanostructured WO_3_ is a well known catalyst, the simultaneous photocatalytic degradation of organic pollutants present in the separated water from the oil water separation process were tested using WO_3_ coated surfaces under UV radiation and the efficiency of this degradation was found to be quite significant. These results assure that with little improvisation on the oil water separation system, these surfaces can be made multifunctional to work simultaneously for oil-water separation and demineralization of organic pollutants from the separated water. Fabrication of the separating surface, their morphological characteristics, wettability, oil water separation efficiency and photo-catalytic degradation efficiency are enunciated.

## Introduction

In the age of ever growing industrialization and energy consumption, the mixing of crude oil in sea water and industrial effluents laden with oil components/pollutants are becoming more common and these adversely affects precious water bodies. Reckless human activities such as discharging enormous tons of oil in the sea during wartime, oil industry accidents and various gallons of water from oil fields processes have all contributed to environmental disasters^1–6^. One of the major challenge faced by environmental researchers and managers in the oil industry is to find out an effective method for oil water separation in order to comply with the stringent national and international environmental regulations. There are many established systems for oil water separation based on physical, chemical and biological methods like API (American Petroleum Institute) oil water separator, vertical gravity separator and ultrasonic separator, separation using electric field, coagulation and biological treatments. These methods of oil water separation need cumbersome costly instrumentations and still most of these processes yield low oil water separation efficiency and lead to the generation of new kinds of pollutants as byproducts. Also, various membrane-based oil water separation techniques have been tried and still the major drawbacks of these methods of separation are low efficiency, rapid fouling up of membrane and the difficulty in the disposal of the contaminated membranes^7, 8^.

Since oil-water mixture is basically an interfacial phenomenon, an effective oil-water separation method can be developed by harnessing the wetting properties oil and water on the material coated on the adsorbing or separating medium. By using a material which has preferential wetting characteristics (i.e. wettable by either oil or water phase and non-wettable by the other), one phase from the mixture can be removed and the other is retained. For example, the materials with hydrophobic-oleophilic in nature are wettable by oil and non-wettable by water and the materials with this kind of wettability have been proposed as effective media for oil removal by adsorption^9–12^. Also hydrophobic-oleophilic material can easily be prepared by modifying the surface energy of the material to be smaller than the surface tension of water and larger than the surface tension of oil^13–17^. However, the limitation of oil water separation using such material as adsorbent is that after adsorbing a certain amount of oil, the surface saturates and the adsorbing efficiency significantly goes down and hence only a small quantity of oil can be separated. Also the used material needs to be recycled for further use or discarded; else the materials become secondary pollutant. Also this method will not be cost effective if the fabrication of the material is expensive and needs to be replaced after every single use. Another method for oil water separation is based on a separation membrane where hydrophobic-oleophilic material is coated on porous material such as fabric or mesh and in this separation method oil phase permeates through the membrane and the water phase is retained^16, 17^_._ However, the inherent limitation of this separation technique is that since the specific gravity of the oil is smaller than that of the water, after some time, the water phase tends to accumulate and forms a column in the lower part and this blocks the oil phase from permeating.

Another oil water separation method that could be contemplated employ the materials with opposite wetting properties of the one discussed earlier. This method is a gravity driven oil-water separation, where the separating medium is a porous surface like a stainless steel mesh coated with hydrophilic-oleophobic material, (wettable by water and non-wettable by oil). With these kinds of composite surfaces, water is expected to permeate through the mesh, while retaining the oil, and also since the material is wettable by water, the water permeates naturally through the mesh without any external forces. Also, this type of material can respond to external trigger/parameter such as electric field, temperature, humidity, and the pH of the environment^19–21^ and hence can open a new avenue for controllable oil-water separation. However, the materials with this kind of wettability are very rare and difficult to synthesize^18^, and even theoretically this kind of surface cannot be realized by lowering the surface energy of the materials. In the case of oil water separation methods, using both hydrophobic-oleophilic and hydrophilic-oleophobic meshes, work on the principle that the capillary force of the wetting liquid phase points downward and that of the non-wetting liquid phase points upward and this tends the wetting liquid phase to permeate through the mesh and the non-wetting one tends to be retained.

Due to the technical simplicity and high oil water separation efficiency of these separation methods, many research groups have worked on the development of surfaces with superoleophobic-superhydrophilic wettability. Since some of the synthesis methods are cumbersome (and perhaps not reliable) and costly, researchers have turned their attention to the possibility of harnessing the properties of superomniphilic (wettable by any type of liquid) material as an alternative for the application of oil-water separation. Motivated by the superomniphilic wetting behavior of fish scale, Lie Jiang’s Group in 2009 discovered the new kind of material for gravity driven oil-water separation^22^. He observed that fish scale is superomniphilic in air and becomes an extremely oil repellent (underwater superoleophobic surfaces), when immersed in water and pointed out that this extreme underwater oil repellency is due to the unique surface structure of the fish scale which possesses a double scale roughness (i.e. micro and nano scale). The trapped water within the surface roughness creates a solid-liquid composite interface, which is repulsive to oil and with this idea in mind, he prepared hydrogel coated stainless steel mesh for oil water separation, which is superhydrophilic and superoleophobic in water^23^. After this finding, many research works have been conducted to harness the underwater oil repellency of materials for oil-water separation and the results were very successful and showed high performance and high separation efficiency^24–29^.

In this work, we adopted a simple, rapid, inexpensive and scalable method to fabricate superhydrophilic-underwater superoleophobic surfaces by spray coating commercially available nano structured ZnO and WO_3_ on the stainless steel meshes. The surface morphology for surface roughness and wetting characteristics of the coated surfaces were studied and these coated surfaces were used as a separating medium for the gravity driven oil water separation. The coated meshes exhibited an excellent water affinity and strong underwater−oil repellency and consequently the oil water separation system showed the separation efficiency of more than 99% for all the oil –water mixtures used for membranes of different pore sizes and coated materials. The comparative merits of nano structured WO_3_ and ZnO coated stainless steel meshes of different pore sizes, in terms of their efficiencies in the process of oil water separation were studied in the light of morphology and surface wettability of the fabricated surfaces. The wettability study of both surfaces indicated that the WO_3_ coated stainless meshes have better potential than ZnO coated stainless meshes for oil-water separation, as the former is capable of working with meshes of larger pore sizes. The motivation of using nano structured WO_3_ as the coating material is that, besides providing adequate surface roughness for underwater oil repellency, they can act as a photocatalyst for the photocatalytic disinfection of organic pollutants and microorganisms present in the permeated water with a simple UV/VIS light or solar radiation. Also in this work we demonstrate the capability of these coated surfaces to photo degrade organic pollutants present in the separated permeate of the oil water separation, which affirms the prospect of using these surfaces as multifunctional for simultaneous oil water separation and the photo degradation of organic pollutants in the filtrate. It was reported that there is a prospect of improving the photocatallytic efficiency of nanostructured WO_3_ by doping them with noble metals like palladium for the photocatalytic disinfection and purification of water^30^. The novelty in using WO_3_ coated mesh for oil water separation is that these surfaces have the potential to work as the multifunctional membrane, where the oil water separation and photocatalytic water purification can be simultaneously carried out. This is because WO_3_ is a proven photocatalyst for the efficient photocatalytic degradation of organic pollutants and microorganisms like sulfide reducing bacteria present in the crude oil^31, 32^. Also this photocatalytic efficiency of WO_3_ can further be improved by doping it with a noble metal like palladium^33, 34^. To the best of our knowledge, WO_3_ coated membrane for simultaneous oil-water separation and photocatalytic degradation of pollutants has not been reported. Another novelty in using WO_3_ coated stainless steel mesh is that it showed high separation efficiency (99%), with the stainless steel mesh of pore size as high as 150 µm, whereas ZnO coated surfaces failed in the process of oil separation with the pore size larger than 50 µm at the same intrusion pressure. In addition to this, the method of fabrication of the coated surface by spray coating is quite simple, cost effective, fast and can be easily extended to larger surfaces.

## Results and Discussion

By the simple method of spray coating, the nanostructured ZnO and WO_3_ particles were deposited on the stainless steel meshes of different pore dimensions. The coated surfaces showed high level of water affinity in air and oil repellency in water and these surfaces were applied for the separation of oil and water in the oil-water mixture. The fabrication of the coated membrane by one step spray coating is described in the experimental section. It was observed that the coated surfaces with appropriate pore sizes showed high efficiency of oil water separation, contrary to the uncoated meshes, through which the oil water mixture just passed without separation. As we discussed in the introductory section, the surface roughness is instrumental in bringing about the underwater oil repellency and hence the study of surface morphology and the consequent changes on various contact angles of the coated surfaces are very important. In the following sub sections, we present the morphology, wettability and oil-water separation efficiencies of both nanostructured ZnO and WO_3_ coated surfaces and discuss their relative merits based on different surface parameters.

### Surface Morphology of Nanoparticles Coated Mesh

**Figure 1:**
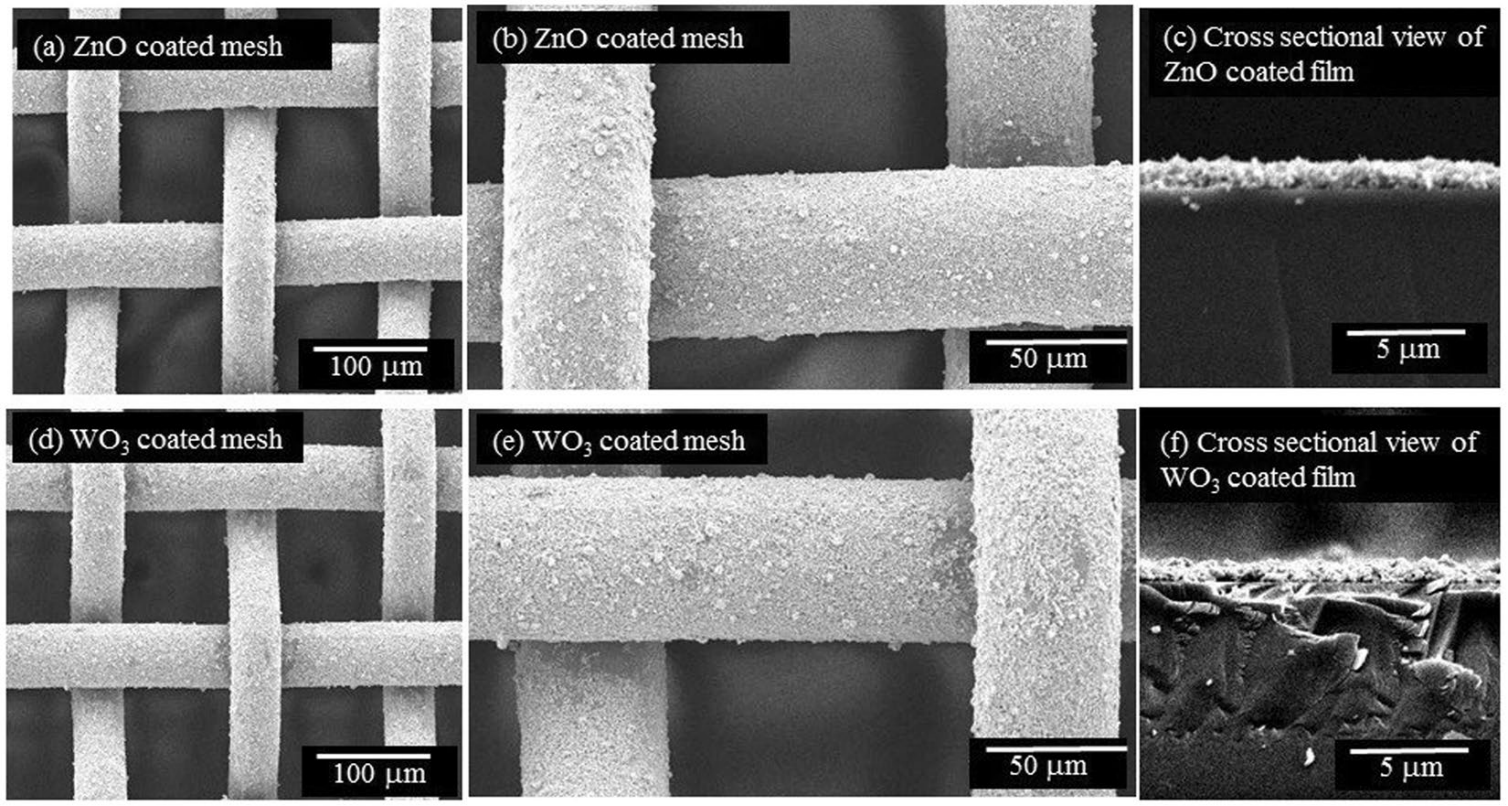
SEM image of coated stainless steel mesh with the pore size of 100 µm, ZnO coated mesh at (**a**) 200 X (**b**) 500 X magnification and (**c**) cross sectional view of ZnO film at 4000 X magnification. WO_3_ coated mesh at (**d**) 200 X (**e**) 500 X magnification and (**f**) cross sectional view of WO_3_ film at 4000 X magnification. Both and WO_3_ and ZnO coated surfaces have a uniformly distributed granular structure with micro and nanoscale hierarchical roughness and free from agglomeration. The cross sectional views further clarify the uniform distribution of the nano particles with the average thickness of 1 to 2 microns.

The SEM images of ZnO and WO_3_ coated stainless steel meshes were taken to study their surface morphology. Figure 1a and b show the SEM images of ZnO coated stainless steel mesh of 100 µm pore size with different magnifications along with the cross sectional view of the ZnO coated surface (Fig. 1c). Similarly, Fig. 1d and e show the SEM images of nano structured WO_3_ coated on the stainless steel mesh with different magnifications and the cross sectional view of WO_3_ coated surface (Fig. 1f). It is quite evident from Fig. 1, that the surfaces coated with both ZnO and WO_3_ nanoparticles have a uniformly distributed granular structure, less clustered agglomeration and micro and nanoscale hierarchical roughness. The cross sectional views shown in Fig. 1c and f, further clarify that the nano particles look uniformly distributed to the whole surface with the average thickness of 1 to 2 microns. Based on Young−Dupré equation^35^, hydrophilic flat surfaces in air can become oleophobic under water.

1 $${\gamma }_{ow}\,\cos \,{\theta }_{ow}={\gamma }_{wa}\,\cos \,{\theta }_{wa}-{\gamma }_{oa}\,\cos \,{\gamma }_{oa}$$

where γ_ow_, γ_wa_ and γ_oa_ are the interfacial tensions of oil-water, water-air and oil-air respectively. From equation 1, it is quite clear that the value of *θ*_*ow*_ becomes high when *γ*_*wa*_ cos *θ*_*wa*_ is greater than *γ*_*oa*_ cos *θ*_*oa*_, which clearly indicates that by increasing the hydrophilicity of solid surface, the underwater oil repellency can be improved^36^.

**Figure 2:**
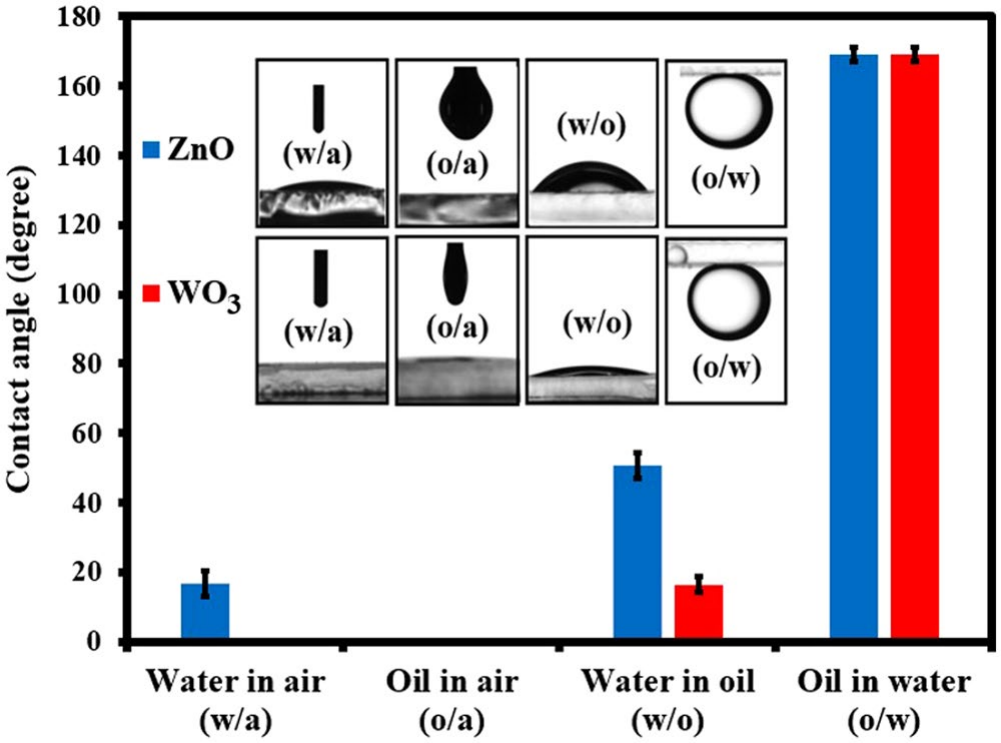
Contact angle images and measurement of ZnO and WO_3_ films deposited onto glass: water in air (w/a), oil in air (o/a), water in oil (w/o) and oil in water (o/w). The water air contact angle θ_wa_ for ZnO is non zero unlike in the case of WO_3_ film and also water in contact angle θ_wo_ for ZnO is much higher than that of WO_3_ and these facts suggest that WO_3_ films have better hydrophilicity than ZnO films.

It is obvious from Fig. 1 that the coated surfaces are quite rough with cavities, and when the water comes in contact with this surface, it fills all the cavities on the surface and forms a new composite solid-oil-water interface and this new interface exhibits underwater superoleophobic properties quite analogous solid-oil-air interface exhibiting superoleophobic nature in air. The underwater superoleophobicity is brought about by the trapped water in the cavities of the surface structures by preventing the permeation of oil droplets and this water membrane can selectively separate water from oil-water mixtures under gravity. Another advantage is that oil fouling of the membrane is minimized due to the low affinity of oil droplets on the membrane under water. In addition to the underwater oil repellency due to the nature of the coated material, the surface morphology and roughness also contribute to enhance the oil repellency which is evident from the following Cassie-Baxter equation shown in equation 2 ^37, 38^.

2 $$\cos \,{{\theta }_{ow}}^{CB}={r}_{f}\,f\,\cos \,{\theta }_{ow}+f-1$$

where *r*_*f*_ is the ratio of the contact line of the portion of the surface, which is in contact with oil and the real contact line and similarly *f* is the projected area of solid surface in contact with oil. In the oil/water/solid system, the micro/nano structure on the surface plays an important role to bring about a Cassie state, where the water molecules are trapped inside the microstructure of the surface to form a composite water-solid interface and this trapped water inhibits the penetration of oil and the surface becomes under water superoleophobic. All the coated surfaces of both ZnO and WO_3_ were annealed at 550 °C and the sole purpose of annealing the coated surface was to improve its mechanical robustness. It is quite noticeable from the SEM images (See Supporting information: Figures S1 and S2), although annealing made the crystal sizes larger in both cases, the required micro and nano structures are still present and consequently, WO_3_ and ZnO coated meshes exhibit high oil under water contact angles, and very small sliding angle as shown in Fig. 2. Hence, it is quite obvious that the annealing of the coated membranes does not affect the performance of oil water separation.

### Wettability of WO_3_ and ZnO Coated Films

The measurements of contact angles and sliding angles (*θ*_*sa*_) were carried out to study the wetting properties of WO_3_ and ZnO coated surfaces and the results are shown in Fig. 2. The inset in Fig. 2 show the contact angle measurement for the WO_3_ and ZnO coated surfaces for four different interfaces: water in air (w/a), oil in air (o/a), water in oil (w/o), and oil in water (o/w).

From Fig. 2, it is obvious that the wetting property of ZnO films is slightly different from that of WO_3_ films, where we can observe that the value of *θ*_*wa*_ is non zero for ZnO unlike in the case of WO_3_ film and also the value of *θ*_*wo*_ for ZnO is much higher than that of WO_3_ films. These two facts suggest that WO_3_ films have better hydrophilicity than ZnO films. Although the value of *θ*_*ow*_ for ZnO is comparable to that of WO_3_, the measurement of oil in water sliding angle shows that there is a small difference in terms of their oil wettability under water, where the sliding angle for WO_3_ coated glass is 1.2 ± 0.4°, and the same for ZnO coated glass is 2.1 ± 0.9°. It was reported that a surface being underwater-oil repellent alone does not guarantee that the coated mesh is a good membrane for oil-water separation^39^. Besides this, the water repellency on the oil surface is also an important factor to channel the water through the mesh during oil-water separation. In order to study this, the WO_3_ and ZnO coated glass slides were immersed in hexadecane and a water droplet of approximately 6 ± 1 μL volume was placed on the coated surface and the water/oil (or w/o) contact angle was found to be *θ*_*wo*_ ~ 16.4 ± 2.1° and *θ*_*wo*_ ~ 50.7 ± 3.6° respectively for WO_3_ and ZnO. This result confirms that even in the hexadecane oil environment, although both the surfaces show good water affinity on oil surface, water wets better on the WO_3_ coated surface than on the ZnO coated surface. Also we infer that water replaces the oil trapped in the porous surface more in the case of WO_3_ coated surface than in the case of ZnO coated surface. This replacement of oil by water on the surface accounts for the process of water channeling through the mesh while retaining the oil during oil−water separation. These high values of *θ*_*ow*_ and small values of sliding angle indicate that the WO_3_ and ZnO coated meshes have the potential for oil-water separation application.

### Performance of Oil Water Separation

**Figure 3:**
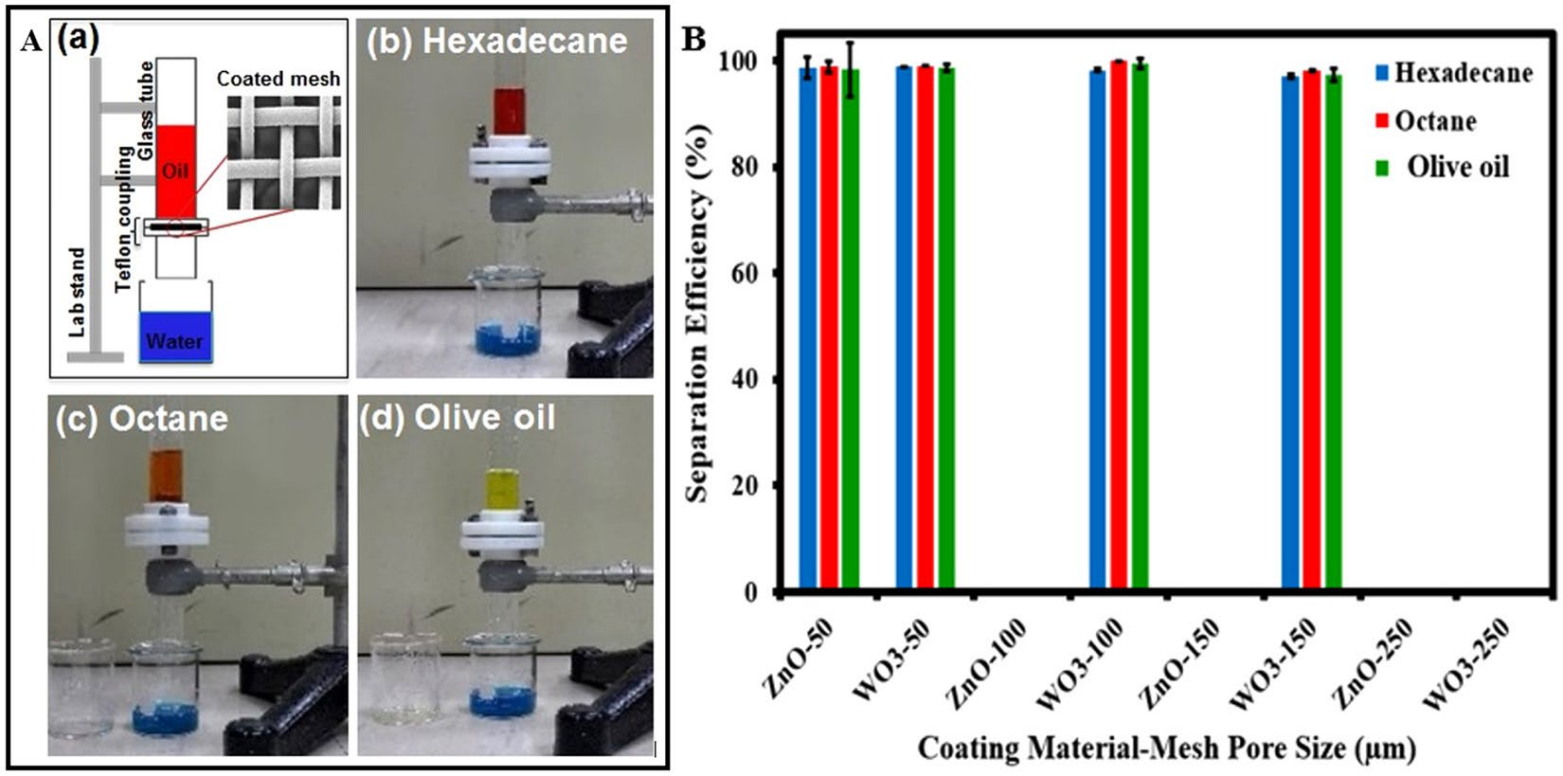
(**A**) (a) Schematic diagram of oil-water separation system, (b), (c) and (d) are pictorial views of oil−water separation setup using WO_3_ coated mesh (100 micron pore size) for hexadecane, octane and olive oil respectively. The oils are colored with the red/yellow, while water is colored with the methylene blue dye. As a result of the exceptional underwater superoleophobic behavior of the WO_3_ coated mesh, water passed through these meshes while retaining oil above and we observed that the permeated water in this process is practically free from any traces of oil and also the filtration process was quite fast. (**B**) The separation efficiency of ZnO and WO_3_ coated meshes with pore sizes of 50, 100, 150 and 250 µm for hexadecane, octane and olive oil. Both WO_3_ and ZnO the coatings show about 99% efficiency in oil water separation, the WO_3_ coated membrane is usable with the stainless steel mesh pore size up to 152 µm, while maximum pore size for ZnO coated mesh can handle is only 50 µm. In the case of ZnO coated membrane, when the pore size above 50 µm was used, the oil water separation failed while, in the case of WO_3_ coated membrane, the oil-water separation is possible for the mesh of pore size as large as 150 µm.

The oil water separation system used in our work is quite simple and basically two glass tubes separated by WO_3_ and ZnO coated meshes (serving as a membrane) and is held together by Teflon flange as shown in Fig. 3A. Before pouring the oil-water mixture, the separating membrane was pre-wetted with water, which is an important step for the successful oil-water separation. The oils used to test our oil-water separation system were hexadecane, octane and olive oil. The oils were colored red (olive oil shows its natural yellow color), and water is colored with the methylene blue dye. As a result of the exceptional underwater superoleophobic behavior of the WO_3_ and ZnO coated meshes, oil was repelled by the surface and consequently the water passed through the mesh and the oil was retained. We observed that the separation process was quite fast and also the permeated water was practically free from any traces of oil (See supporting information: Videos S1, S2 and S3). Figure 3A show pictorial views of oil−water separation setup using WO_3_ coated mesh (100 micron pore size) for three different oils (hexadecane, octane and olive oil).

In order to prove the effectiveness of our coated surfaces in the oil-water separation process, initially a uncoated SS mesh was used in between the glass tubes and with this membrane in place, both the oil and water permeated through the mesh resulting in the failure of oil water separation (Supporting information: Figure S3). But when the coated mesh was used as a separating membrane in between the glass tubes, the membrane surface permeated only water and retained oil on the top as shown in Fig. 3(b–d). In this work the efficiency of the oil-water separation is conveniently estimated using the following equation^40, 41^.

3 $${Efficiency}({ \% })=\,[1-\,\frac{{C}_{P}}{{C}_{O}}]\times 100$$

where C_o_ and C_p_ are the measured volume/volume proportions (v/v) of the oil and water in the initial mixture and the permeate respectively.

Figure 3b shows the oil water separation efficiency estimated using equation 3 for three different oils (hexadecane, octane and olive oil) with ZnO and WO_3_ coated meshes of different pore sizes. Although membranes coated with both ZnO and WO_3_ achieved 99% efficiency in oil water separation, it was observed that the WO_3_ coated membranes are usable with the stainless steel mesh of pore size up to 150 µm, while maximum pore size that a ZnO coated membrane can withstand is only 50 µm. In the case of ZnO coated membrane, when the pore size above 50 µm was used, both oil phase and water phase permeated through the mesh resulting in the failure of the separation process, while in the case of WO_3_ coated membrane, the oil-water separation is possible for the mesh of pore size as large as 150 µm. A further investigation is needed to understand the correlation between the nature of the coated material and the maximum pore size that the membrane can be successful. Presumably this correlation between the coated material and the maximum sustainable pore size could be due to some kind of adhesive force between the coated material on the rim of the pore and water molecules, which decides the maximum sustainable pressure created by the oil drop. From this result, we conclude that, compared to ZnO coated membranes, WO_3_ coated membranes are better for oil-water separation application.

### Mechanism of Oil Water Separation

**Figure 4:**
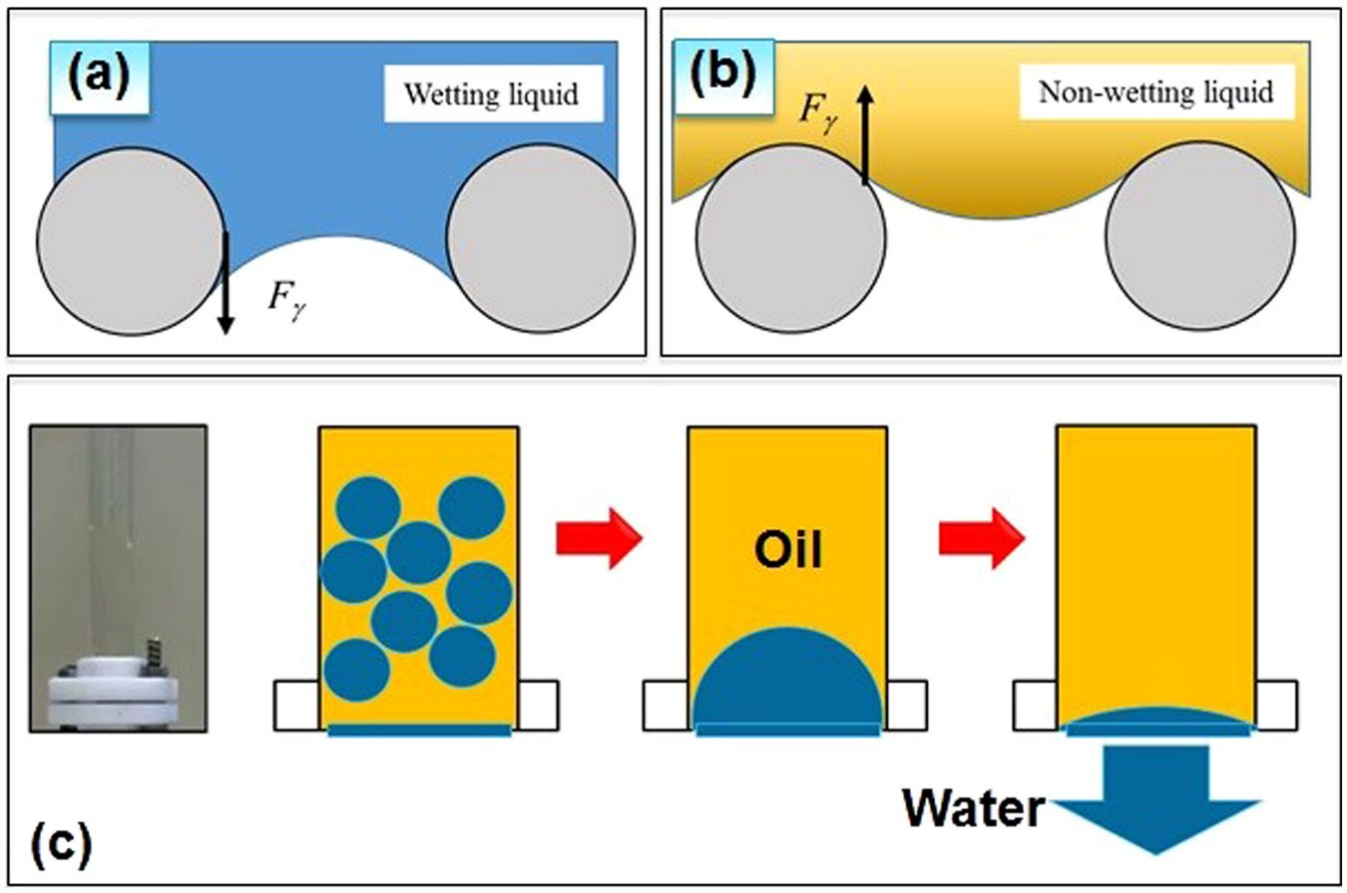
(**a**) The direction of the capillary force (F_γ_) of wetting liquid phase, (**b**) The direction of the capillary force (F_γ_) of Non-wetting liquid phase and (**c**) the suggested mechanism of oil-water separation due to the opposite directions of the capillary forces of wetting water and non-wetting oil phases. The oil water separation is possible because of the difference in the direction of the capillary forces for oil and water. The water film has role as a barrier to prevent the oil from penetrating the mesh. When the oil phase comes in contact with the coated mesh, it will be sustained by the capillary force of oil in which the direction is the opposite of the weight force. On the other hand, when water phase comes, it will be diffused with the water layer and transferred to the other phase of the mesh.

As mentioned earlier, the kind of oil water separation described in this work, the capillary forces between the two phases of liquids and solid surface act in the opposite directions, where, the direction of the capillary force (F_γ_) of wetting liquid phase points downward, and the same for the non-wetting liquid phase points upward as illustrated in Fig. 4a and b. When the oil water mixture is poured in the upper tube, two mechanisms take place: (i) due to the superhydrophillicity of the surface, the water comes in contact with the coated surface on the wires of the mesh and (ii) if the pore size of the mesh is smaller than the capillary length of water, a water film is formed between the wires of the mesh. The formation of a water layer is very crucial as it has two important roles in the mechanism of oil water separation: first, due to under water oil repellency, the water film prevents oil droplet to be in contact with the mesh and the second function is that the water film acts as a channel for the water droplets in the oil-water mixture to diffuse to the other side of the mesh. Hence the coated film must be pre-wetted by water to create water film attached between the pores before using it for oil−water separation. The water film has role as a barrier to prevent the oil from penetrating the mesh. When the oil phase comes in contact with the coated mesh, it will be sustained by the capillary force of oil in which the direction is the opposite of the weight force. On the other hand, when water phase approaches, it will be diffused within the water layer and transferred to the other phase of the mesh as illustrated by Fig. 4c. In summary, the oil water separation is possible because of the difference in the direction of the capillary forces for oil and water.

**Figure 5:**
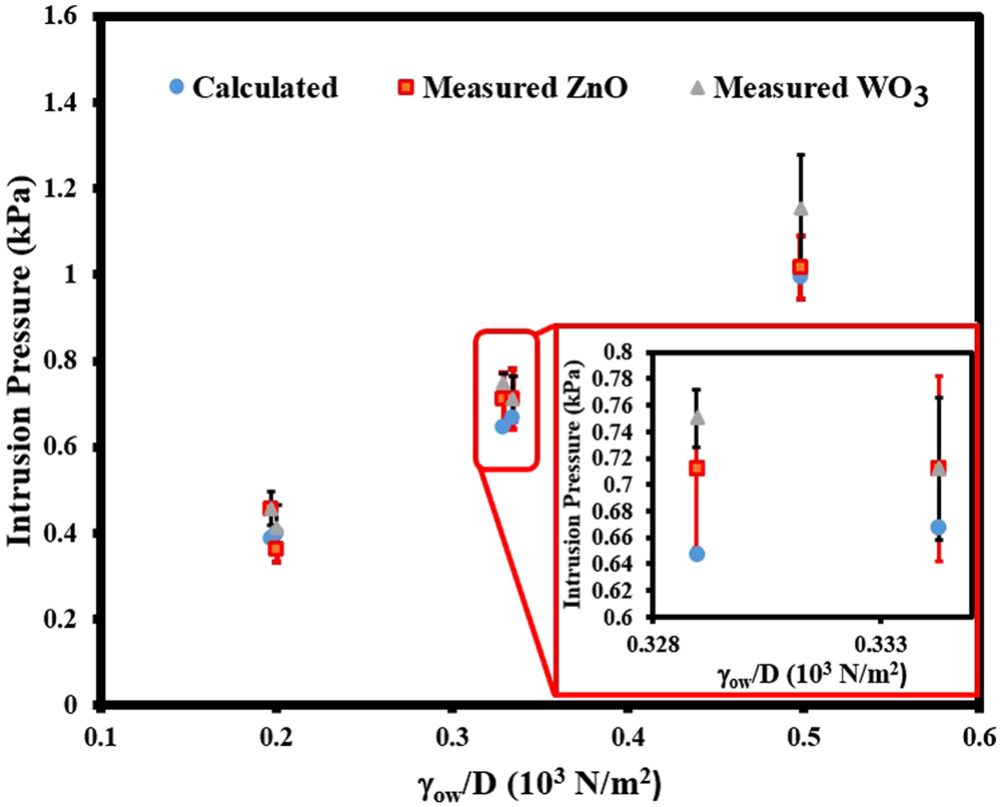
The intrusion pressure of ZnO and WO_3_ coated mesh with different pore sizes. The measured value was determined by measuring the maximum hydrostatic pressure and the calculated value was obtained using equation in the manuscript. From these graphs, we can see that the experimental data is statistically in agreement with the calculated value.

It was observed that the oil-water separation did not yield a desirable result when the pore size of the stainless steel mesh is more than 50 and 150 micron respectively for ZnO and WO_3_ coated meshes. Also when the height of the liquid column in the tube above the membrane exceeds certain height (*h*_*max*_), the oil- water separation fails, where the oil starts penetrating through the coated mesh. The hydrostatic pressure (*ρ*g*h*_*max*_) corresponds to *h*_*max*_ is equal to the intrusion pressure of the membrane where *ρ* is the density of oil. The intrusion pressure is *P*_*int*_, the maximum pressure that the coated mesh can withstand, which can be theoretically approximated using this equation:

4 $${P}_{int}=-\,\frac{4{\gamma }_{ow}\,\cos \,{\theta }_{ow}}{D}$$

where D is the pore size of the coated mesh and other factors in the equation are as explained earlier. It is clear from the above equation that larger the pore size, smaller the intrusion pressure and this explains the reason for the observed incapability of the coated meshes of larger pore sizes to function as a membrane for oil-water separation. Also the intrusion pressure can be seen as the pressure difference between water phase and the oil phase (*P*_*int*_ = *ΔP* = *P*_*water*_ − *P*_*oil*_), and if the value of *P*_*int*_ is positive, then the pressure of water phase is greater than the pressure of the oil phase, so that oil cannot flow through the pore of the mesh unless an external pressure is applied to overcome the pressure difference. Also, it is quite clear from equation 4 that as long *θ*_ow_ is larger than 90 degree (under water superoleophobicity), the intrusion pressure remains positive and hence the water pressure should be higher than the oil pressure and this prevents oil from flowing down. On the contrary, if the intrusion pressure *P*_*int*_ is negative, oil will channel through the mesh even without the aid of external pressure. Figure 5 is the comparison of the experimental value with the calculated value of the intrusion pressure *P*_*int*_ versus *γ*_*ow*_/*D* for ZnO, and WO_3_ coated meshes. The measured value was determined by measuring the maximum hydrostatic pressure discussed earlier and the calculated value was obtained using equation (4). From these graphs, we can see that the experimental data is statistically in agreement with the calculated value.

It should be noted that equation 4 is applicable only for oil water interface. When the height of the water column reaches *h*_*max*_, the oil phase approaches the bottom line of the water film and this will result in the formation of a new oil water and vapor interface within the pore of the mesh and consequently the contact angle changes. In this situation we can imagine that oil droplet sitting on water in air environment and the new contact angle formed is described using equation (5) (Young’s equation of contact angle).

5 $$\cos \,\theta =\frac{{\gamma }_{wa}-{\gamma }_{{\rm{ow}}}}{{\gamma }_{oa}}$$

Since the value of *γ*_wa_ is generally larger than *γ*_ow_, the value of cos *θ* is positive and consequently, the intrusion pressure *P*_*int*_ becomes negative and this explain the failure of oil water separation through the coated mesh once h_max_ is reached and keeps flowing even after h is made lower than h_max_. The formation of water-film between the wires of the mesh is possible when size of the mesh is much smaller than the capillary length of water^42, 43^. The presence of a water film between the wires ensures the formation of oil water interface and hence equation 4 becomes applicable. Hence, for a successful oil water separation process, it is important to select a porous substrate where a robust capillary bridge can be formed and can sustain high external pressure. Also in the case of continuous flow of the oil-water mixture, it will remain supported indefinitely. However, as the water film formed on the surface is very crucial for this process, the system fails and oil will start permeating through the membrane, when this water thin film on the surface dries out (~after 4 hours). However it has been proved recently that under UV irradiation, such coatings (ZnO, WO_3_) can be effective as antifouling and work for longer periods.

### Photo catalytic Degradation of Organic Dye in the Separated Water

**Figure 6:**
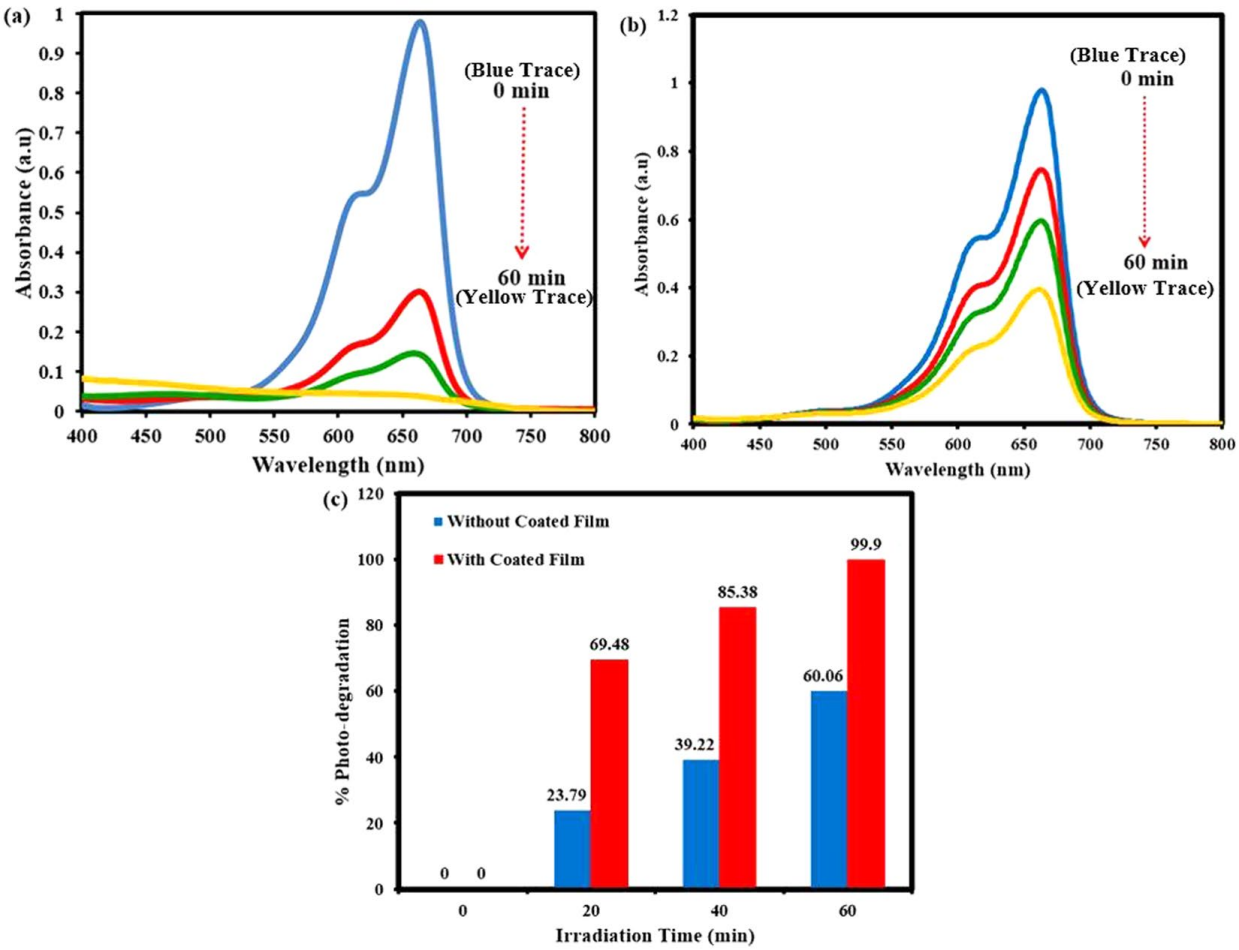
(**a**) Photo-degradation of organic pollutant (MB solution) in the presence of WO_3_ nanoparticles coated film under visible light irradiation and (**b**) depicting the irradiation time dependent photo-degradation efficiency of organic pollutant without WO_3_ nanoparticles coated film. After one hour of UV irradiation with WO_3_ nanoparticles coated surface the photo-degradation is 99.9% compared to 60% of degradation in the absence of the coated surface.

As mentioned earlier, another interesting functionality of our WO_3_ and ZnO coated surfaces is its capability to photo-catalyze the degradation of organic pollutants present in the separated water from the oil water separation process. It is quite well known that the light in general and UV light in particular is capable of degrading almost all organic dyes even in the absence of any photocatalysts. In our study we used this as a bench mark to compare the increased efficiency that the nanostructured WO_3_ coated surface brings about in the process of photo-degradation of organic pollutant (methylene blue dye in water) under UV light irradiation. Figure 6(a) and (b) respectively show the absorption spectra of the methylene blue in water with and without WO_3_ coated surface under UV light irradiation of same energy and irradiation time. The bar chart in Fig. 6(c) shows the percentage of degradation of methylene blue mixed water at different irradiation times, where it is quite obvious that at each irradiation time the presence of coated surface makes a considerable difference in the dye degradation process. As we can see in Fig. 6(c), after one hour of UV irradiation with coated surface the photo-degradation is 99.9% compared to 60% of degradation in the absence of the coated surface. This study demonstrates that with a little technical improvisation this coated surface has the potential of serving as a multifunctional surface for simultaneous oil water separation and photo-degradation of organic pollutants.

## Conclusions

We employed facile spray coating technique to fabricate nanostructured WO_3_ and ZnO coated stainless steel meshes of different pore sizes to be used as the membrane for gravity driven oil water separation. The SEM images and the contact angle measurements testify that these surfaces posses the essential surface roughness and the wettabilty required for the efficient oil water separation. It was noticed that WO_3_ coated stainless steel mesh showed high separation efficiency (99%), with pore size as high as 150 µm, whereas ZnO coated surfaces failed in the process of oil-water separation when the pore exceeded 50 µm size. Also owing to the excellent photo-catalytic nature of both WO_3_ under the UV illumination, our coated surfaces efficiently removed the methylene dye present in the separated water. This gives the hope that with proper improvisation of material synthesis and modification in the oil water separation system, the fabricated surfaces have the potential to function as a multifunctional membrane that can be simultaneously used for oil water separation and for the degradation of organic pollutant present in the water. This present study contributes towards the improvement of advanced innovative oil-water separation materials with possible commercial applications combined with solar irradiation.

## Experimental Procedures

The materials used in this work are commercially available photocatalyst materials. Zinc oxide (ZnO) < 100 nm, and tungsten oxide (WO_3_) < 100 nm were purchased from Sigma Aldrich®. The materials were deposited on stainless steel meshes, glass slides substrate and silicon wafer substrates using spray deposition of nanoparticle dispersion. Nanoparticle form of these materials is necessary for spray deposition since the nozzle of the spray gun used is smaller than 1 mm. We found that a stable dispersion cannot be made using material with grain size larger than 100 µm. In addition, the use of nanoparticle helps on texturing the substrate and obtain larger surface area. Stainless steel meshes with pore size of 50, 100, 150 and 250 µm were purchased from TWP Inc. The samples prepared on glass slides and silicon wafers were used for wettability study and coating thickness estimation respectively, while the sample prepared on stainless steel meshes were used for oil water separation test.

The films fabrication started by preparing the nanoparticle dispersions in tetrahydrofuran (THF). The dispersions were prepared and tested in three different concentrations to find the best concentration to be used in this work. We mixed 0.05 g, 0.10 g and 0.15 g of the nanoparticle in 10 ml of THF to obtain dispersion with concentration of 5 mg/ml, 10 mg/ml and 15 mg/ml. The mixtures were then sonicated for 1 hour to create stable dispersion for the spray coating. THF is an organic solvent which has low viscosity and low surface tension. These properties are required to obtain optimum condition for spray deposition technique. Different solvents, such as ethanol, isopropyl alcohol, acetone, methanol, and water, were examined but only THF, isopropyl alcohol and deionized (DI) water showed stable dispersion after sonication process. In the end, THF was chosen because it has low boiling point (Tb = 66 °C) which is preferable for spray coating process as it make the solvent evaporate readily after the deposition process.

Upon deposition, the substrates were cleaned by rinsing with acetone, isopropyl alcohol and DI water. The substrates were then dried using an air blower until all the remaining water evaporated. The deposition process was done inside a fume hood. The spray gun (McMaster Carr) with nozzle diameter of 0.75 mm was used for the spray coating with the nitrogen pressure of 170 kPa. The distance between nozzle and substrate was fixed at 20 cm and the diameter of the sprayed area was 7 to 10 cm. 10 ml of the dispersion was used to coat each face of a substrate. The stainless steel mesh substrates were sprayed on both faces while for glass slides and silicon wafers only one face was sprayed. After deposition process, the samples were annealed in air at 550 °C for 2 hours. This step is required to increase the robustness of the films attached to the substrates.

Surface morphology of the coated stainless steel meshes was studied from the SEM images recorded on a TESCAN FERA3 field emission scanning electron microscope (FE-SEM). Contact angle were measured by a KRUSS (DSA20X, GmbH Germany) standard drop shape analysis system using 6 ± 1 µL water/oil droplet at room temperature. For a more accurate contact angle measurement the average value of measurements were taken. The set up for oil water separation is basically two Pyrex glass tubes with 15 cm length and 1.7 cm diameter having flanged edges to rigidly hold the coated meshes with proper O–ring. The oils used for testing were octane (Sigma Aldrich), hexadecane (Sigma Aldrich) and olive oil (local market) and in order to distinguish water and oil, the oil was colored with red dye and the water was colored with the methylene blue dye. Another purpose of using methylene blue is to demonstrate the capability of our coated surfaces in the photocatalytic degradation of organic dyes present in the water permeated from the oil water separation system.

## Electronic supplementary material

Supplementary Video 1

Supplementary Video 2

Supplementary Video 3

Supplementary information
