# Supplementary material for: Fabrication and Wettability Study of WO3 Coated Photocatalytic Membrane for Oil-Water Separation: A Comparative Study with ZnO Coated Membrane
Source: Sci Rep. 2017 May 10;7:1686. doi: 10.1038/s41598-017-01959-y (PMC5431880; doi:10.1038/s41598-017-01959-y)
Supplement: Supplementary file 4 — Supplementary information [file 41598_2017_1959_MOESM4_ESM.pdf]

Supplementary Information File

Fabrication and Wettability Study of WO_3 Coated Photocatalytic Membrane for Oil-Water Separation: A Comparative Study with ZnO Coated Membrane.

Mohammed A. Gondal*[1], Muhammad S. Sadullah[1], Talal F. Qahtan[1], Mohamed A. Dastageer[1], Umair Baig[1, 2], Gareth H. McKinley[3]

[1]*Laser Research Group, Physics Department, King Fahd University of Petroleum & Minerals, Dhahran 31261, Saudi Arabia*
[2]*Center of Excellence for Scientific Research Collaboration with MIT, King Fahd University of Petroleum and Minerals, Dhahran 31261, Saudi Arabia*
[3]*Department of Mechanical Engineering, Massachusetts Institute of Technology, Cambridge, Massachusetts 02139-4307, United Sates*

Corresponding authors' email:* ***magondal@kfupm.edu.sa* ***(M.A.Gondal)***

Telephone: +9663-8602351/8603274; *Fax:* +9663-8604281

Legends of Videos:

Video S1: Video of oil-water separation using WO_3 coated mesh (100 micron pore size) for hexadecane. The oil is colored with the red, while water is colored with the methylene blue dye. As a result of the exceptional underwater superoleophobic behavior of the WO_3 coated mesh, water passed through these meshes while retaining oil above and we observed that the permeated water in this process is practically free from the traces of oil and also the separation process was quite fast.

Video S2: Video of oil-water separation using WO_3 coated mesh (100 micron pore size) for octane. The oil is colored with the red, while water is colored with the methylene blue dye. As a result of the exceptional underwater superoleophobic behavior of the WO_3 coated mesh, water passed through these meshes while retaining oil above and we observed that the permeated water in this process is practically free from the traces of oil and also the separation process was quite fast.

Video S3: Video of oil-water separation using WO_3 coated mesh (100 micron pore size) for olive oil. The oil is yellow in color, while water is colored with the methylene blue dye. As a result of the exceptional underwater superoleophobic behavior of the WO_3 coated mesh, water passed through these meshes while retaining oil above and we observed that the permeated water in this process is practically free from the traces of oil and also the filtration process was quite fast.

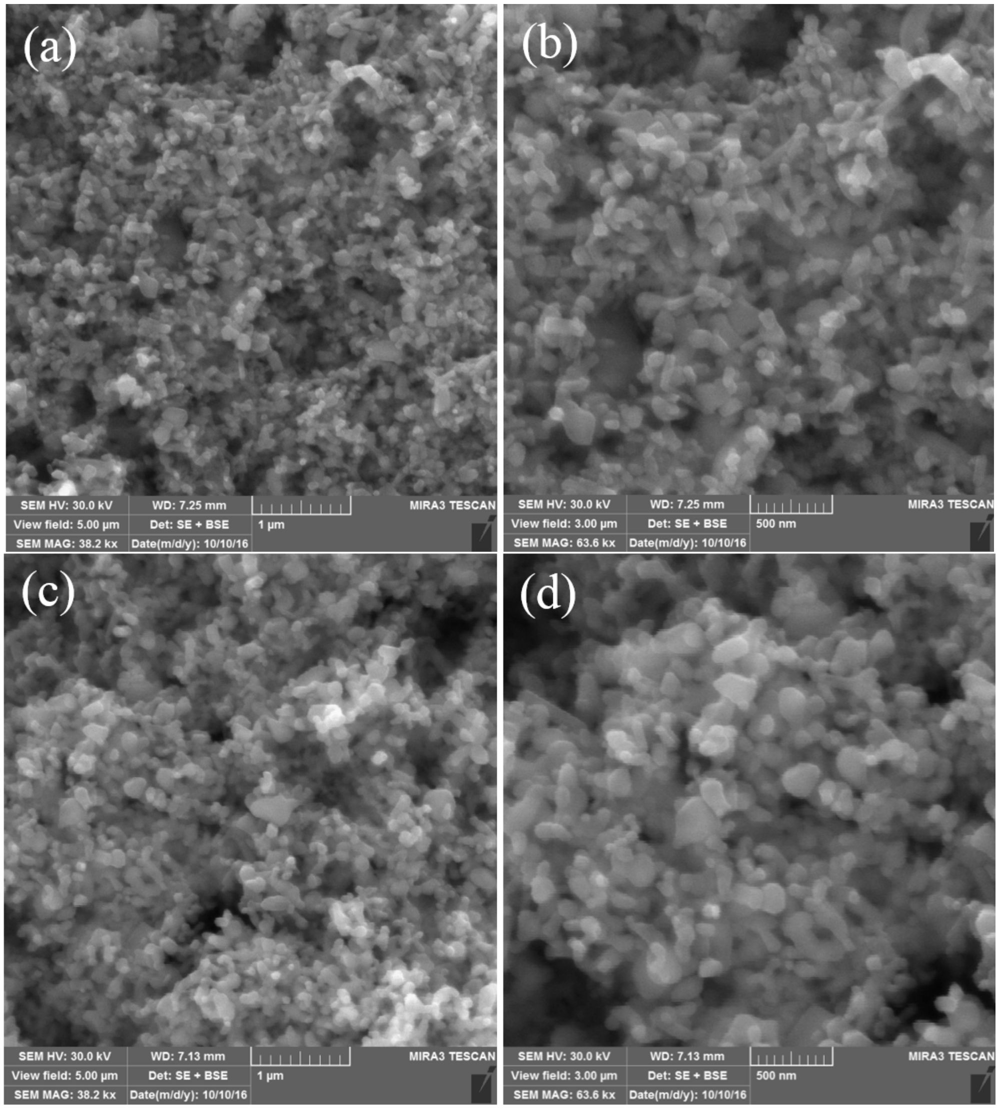

Figure S1: FE-SEM images of (a-b) ZnO nanoparticles before annealing and (c-d) ZnO nanoparticles after annealing at 550 ^{o}C.

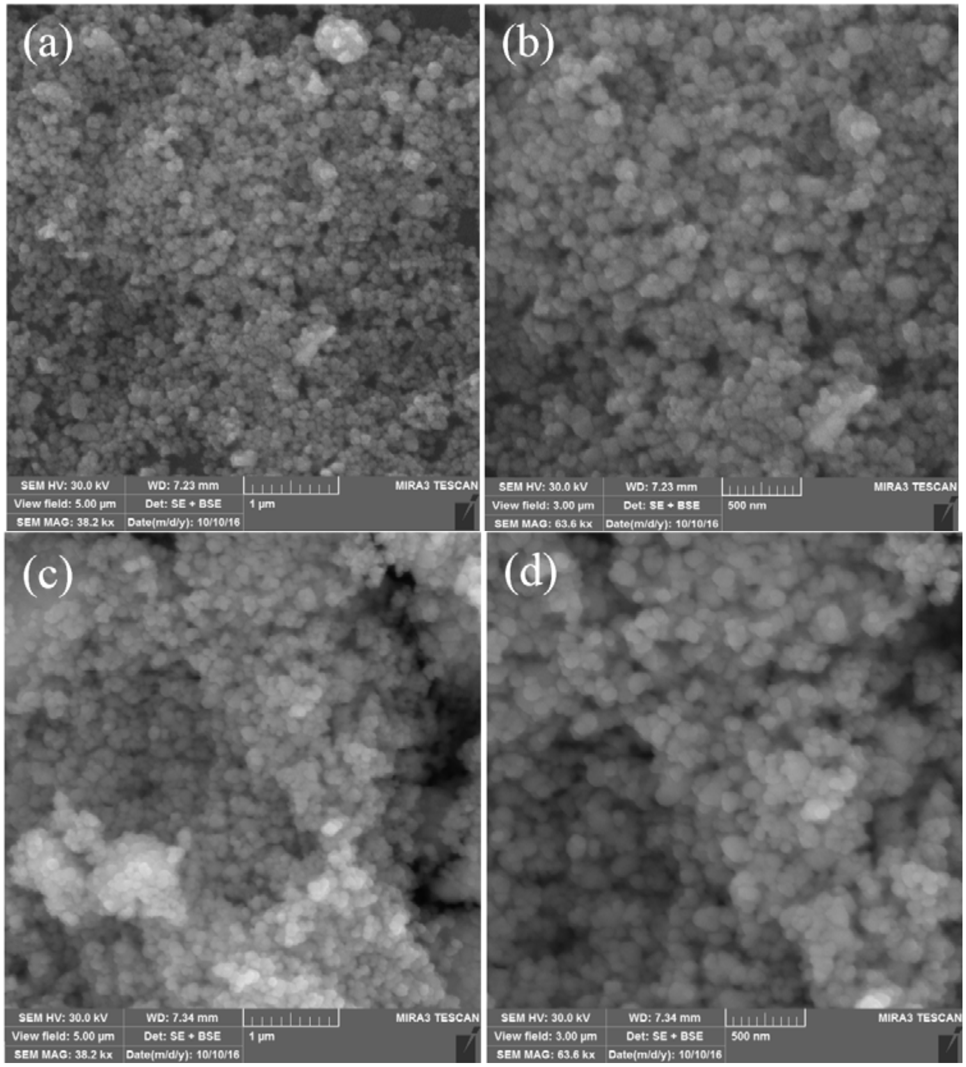

Figure S2: FE-SEM images of (a-b) WO_3 nanoparticles before annealing and (c-d) WO_3 nanoparticles after annealing at 550 ^{o}C.

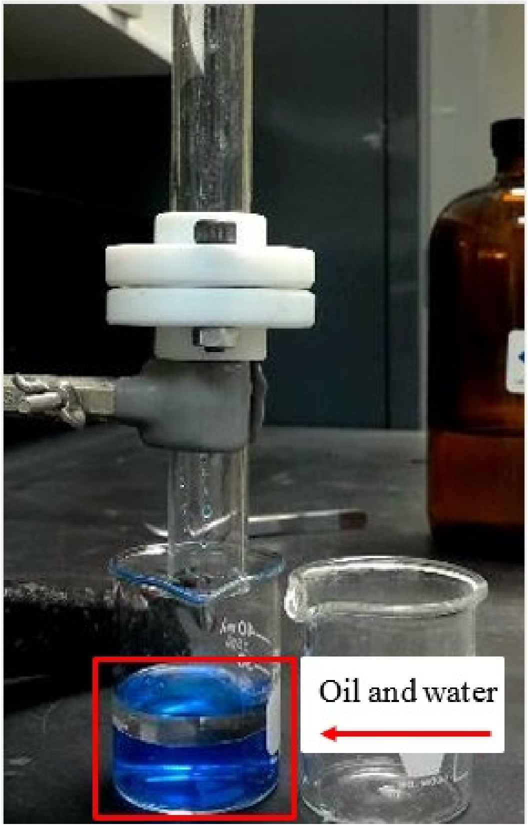

Figure S3: Oil−water separation setup using uncoated stainless steel mesh (100 micron pore size) for hexadecane. Uncoated stainless steel mesh in between the glass tubes and found that both the oil and water permeated through the mesh. The oil (hexadecane) is transparent in color, while water is colored with the methylene blue dye.
